# Antimicrobial Peptides as Anti-Infective Agents in Pre-Post-Antibiotic Era?

**DOI:** 10.3390/ijms20225713

**Published:** 2019-11-14

**Authors:** Tomislav Rončević, Jasna Puizina, Alessandro Tossi

**Affiliations:** 1 Department of Biology, Faculty of Science, University of Split, 21000 Split, Croatia; puizina@pmfst.hr; 2 Laboratory for Aquaculture, Institute of Oceanography and Fisheries, 21000 Split, Croatia; 3 Department of Life Sciences, University of Trieste, 34127 Trieste, Italy; atossi@units.it

**Keywords:** antimicrobial peptides, antimicrobial resistance, AMP identification and design, biosynthesis, mode of action, physico-chemical properties, therapeutic potential

## Abstract

Resistance to antibiotics is one of the main current threats to human health and every year multi-drug resistant bacteria are infecting millions of people worldwide, with many dying as a result. Ever since their discovery, some 40 years ago, the antimicrobial peptides (AMPs) of innate defense have been hailed as a potential alternative to conventional antibiotics due to their relatively low potential to elicit resistance. Despite continued effort by both academia and start-ups, currently there are still no antibiotics based on AMPs in use. In this study, we discuss what we know and what we do not know about these agents, and what we need to know to successfully translate discovery to application. Understanding the complex mechanics of action of these peptides is the main prerequisite for identifying and/or designing or redesigning novel molecules with potent biological activity. However, other aspects also need to be well elucidated, i.e., the (bio)synthetic processes, physiological and pathological contexts of their activity, and a quantitative understanding of how physico-chemical properties affect activity. Research groups worldwide are using biological, biophysical, and algorithmic techniques to develop models aimed at designing molecules with the necessary blend of antimicrobial potency and low toxicity. Shedding light on some open questions may contribute toward improving this process.

## 1. Antibiotics and Antimicrobial Resistance—History is Important

Despite a common misconception, exposure to antibiotics is not confined to the modern antibiotic era starting from the early 20th century. Ancient civilizations used antibiotics to treat bacterial infections, with topical applications of moldy bread to wounds being well documented in ancient Egypt, China, Greece, and the Roman empire [1]. Traces of molecules related to modern day antibiotics, the tetracyclines, have been found in skeletal remains from ancient Sudanese Nubia (350–550 A.D.) and from the Roman period in Egypt [2,3,4]. In both cases, it is presumed that they were ingested with grains contaminated by Streptomycetes [3,4] and most likely had a preventive role rather than a “systemic” effect on infection. Remedies used in traditional Chinese medicine have been the source of potent anti-infective agents for millennia, with artemisinin or “qinghaosu” which is a potent anti-malarial drug, being one of the best known examples [5].

**Figure 1:**
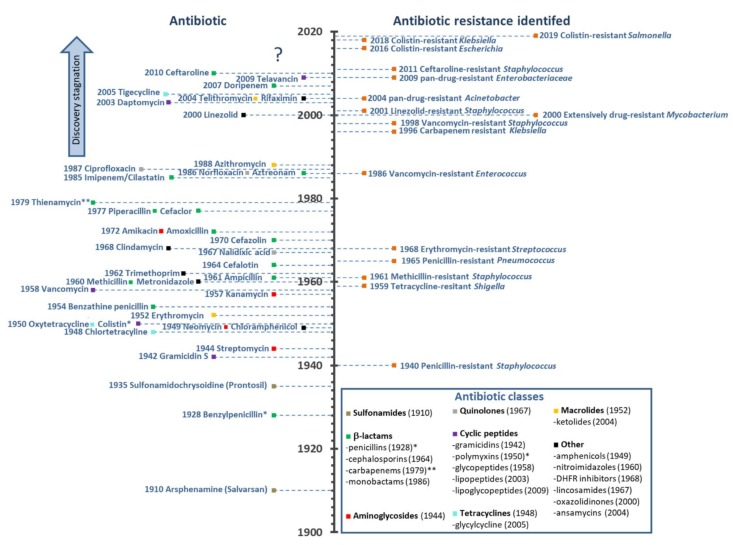
Timeline of antibiotic development as released and in parallel with the timeline for emergence of drug-resistant bacteria [2,6,11,13,17,18,19,20,21,22,23,24,25,26,27,28,29,30,31,32,33,34]. All antibiotic classes are represented by at least one antibiotic. For figure clarity, not all antibiotics nor all antibiotic-resistant bacteria are represented in the timeline. (*) Date of discovery not of release, (**) date of discovery, never introduced due to instability in an aqueous solution [35].

The modern antibiotic era begins with Paul Erlich’s concept of the “magic bullet” at the beginning of the 20th century. Together with the chemist Alfred Bertheim and bacteriologist Sahachiro Hata, he discovered the arsenic dye arsphenamine, which was later called Salvarsan or 606 (it was the 606th compound tested) as a potent anti-syphilis drug [2,6]. This was known as antimicrobial chemotherapy, and the first widely available antibiotic, introduced in 1935 by Gerhard Domagk, was sulfonamidochrysoidine or Prontosil, with antibacterial activity occurring in a number of infectious diseases. It was soon determined that Prontosil is a precursor to *p*-aminophenylsulfonamide, already discovered in 1908 and not patentable, but which led to easily modified derivatives and the era of sulfonamide antibiotics [7]. Penicillin, which is one of the best known antibiotics, was discovered in 1928 by Alexander Fleming, even though mass production started more than 15 years later during World War II, following synthesis and purification work carried out by Howard Florey and Ernest Chain [2,8]. In 1944, Selman Waksman (considered “the father of antibiotics” and who first coined the term) discovered an aminoglycoside antibiotic from *Streptomyces griseus* and named it streptomycin [9,10]. This marked the beginning of a golden age, which led to the discovery of more than 20 different groups of antibiotics in the following decades, of several different types: *sulfonamides*, ***β**-lactams* (with penicillin, cephalosporin, monobactam and carbapenem subclasses), *aminoglycosides*, *quinolones*, *cyclic peptides* (including gramicidins, polymyxins, glycopeptides, lipopeptides and lipoglycopeptides), *tetracyclines* (including glcyclines), *macrolides* (including ketolides), *amphenicols*, *nitroimidazoles*, *dihydrofolate reductase* (*DHFR) inhibitors*, *lincosamides*, *oxazolidinones*, and *ansamycins* (see Figure 1).

There is no doubt that antibiotics have changed the course of medicine and saved untold millions of lives worldwide since they were first introduced. Infectious diseases that could not be previously treated, and their catastrophic effects, could now be easily controlled. However, resistance started to emerge rapidly. Resistance of *Staphylococcus* to penicillin had already emerged by 1940, before mass production had even begun, while resistance toward methicillin was first reported only two years after its introduction (see Figure 1). Fortunately, during the golden age, novel antibiotics kept being discovered and developed for clinical use. The glycopeptide vancomycin, in use from 1958, was widely believed to be resistance-proof, but, by 1986, a resistant *Enterococcus* had appeared, and resistant staphylococci emerged before the end of the century (see Figure 1). Multi-drug-resistant (MDR) bacteria have, by now, become a major concern, with the emergence of pan-drug resistant (PDR) or extensively-drug resistant strains (XDR, see Figure 1) such as *Mycobacterium tuberculosis* resistant to fluoroquinolones and all second-line injectable drugs (capreomycin, kanamycin, or amikacin) [11]. It is conservatively estimated that, in the US and Europe, 2.5 million people are affected by such infections each year, and approximately 50,000 people die as a result of the infection [11]. 

A related major concern is the drying up of the pipeline over the last 30 years, and novel classes of antibiotics entering into it (see Figure 1). Among the limited number of promising compounds at various stages of clinical investigations and development [12], few, if any, represent a truly new class, with a completely different mode-of-action to previously approved drugs. Such an example are oxazolidinones, with linezolid being introduced in 2000, but, unfortunately, staphylococcal resistance emerged shortly after (see Figure 1) [13]. In this period, ketolides, glycylcyclines, and lipopeptides have also been introduced (see Figure 1). However, telithromycin (ketolide) and tigecycline (glycylcycline) showed adverse side effects, which prompted the Food and Drug Administration (FDA) to label the products with their strongest form of warning known as the “black box warning” [14,15]. As a consequence, telithromycin was discontinued from further use. Daptomycin, which is a lipopeptide, was introduced in 2003 and showed success, but was removed from the World Health Organization (WHO) List of Essential Medicines in 2019 [16].

The drying pipeline has resulted in some shelved antibiotics returning to clinical practice despite inadequate pharmacological properties, such as colistin (polymyxin E). Discovered in 1950 by Japanese researchers [21], it was abandoned shortly afterward due to its nephrotoxicity [36] and the abundance of other equally potent antibiotics with less pronounced side effects. It is now being used as a “drug of last resort” against Gram-negative bacterial infections [37]. However, cases of resistance to it have recently been reported [38], with plasmid-mediated dissemination of the *mcr-1* gene was reported in *Escherichia coli* in 2016 [24], of *mcr-8* in *Klebsiella pneumoniae* [25], and *mcr-9* in *Salmonella enterica* [26] soon after (see Figure 1).

It is, therefore, evident that novel anti-infective agents with alternative modes of action are urgently required to battle the ever evolving, multi-drug resistant bacteria. In 2017, the WHO posted a shortlist of the critical strains to combat: carbapenem- and 3rd generation cephalosporin-resistant *Acinetobacter baumannii*, *Pseudomonas aeruginosa*, and *Enterobacteriaceae* [39]. Many research groups worldwide are now devoted to solving this crisis and, among other molecules being considered [40,41], antimicrobial peptides (AMPs) are hailed as possible alternatives to conventional antibiotics for some therapeutic uses, which have a relatively low potential to elicit resistance [42]. Few believe that completely resistance-free antimicrobials can be developed any more.

## 2. Antimicrobial Peptides—What Are They?

“*I Like the Dreams of the Future Better than the History of the Past.*”

-Thomas Jefferson (1743–1826)

**Figure 2:**
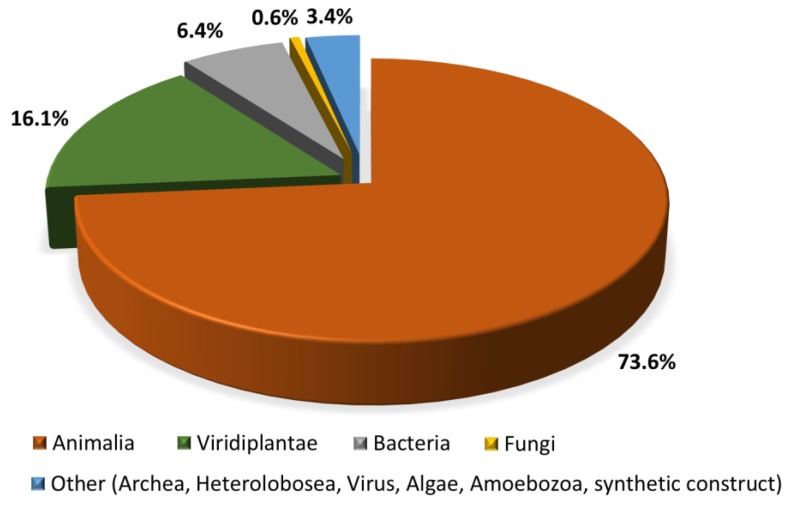
Distribution of AMPs across kingdoms based on sequences in the CAMP^R3^ database. (http://www.camp.bicnirrh.res.in/dbStat.php) [48].

There is no univocal answer to this question, but we can find a consensus of what the antimicrobial peptides research community says that they are: multifunctional effector molecules, often gene encoded, produced by almost all organisms, and having a direct antimicrobial activity and/or immunomodulatory properties [43,44]. In light of the burgeoning resistance problem, peptides with a direct antimicrobial activity (AMPs) and often immune-regulatory capacities are easy to fit to the “dream (molecules) of the future” definition, to paraphrase Thomas Jefferson. Many AMPs have been discovered to date (see below), and they are reported to be active against Gram-negative and Gram-positive bacteria, against infective fungi and sometimes with antiviral, antiparasitic, or antiprotozoal properties [45]. Many of these have also been shown to modulate host immunity by activating immunocytes, and modulating inflammation, alternatively suppressing or promoting it [46]. To emphasize their pleiotropic nature in higher organisms, natural AMPs are often referred to as ‘*host defense peptides*’ (HDP), or, more specifically, as ‘*innate defense regulatory* (IDR) peptides,’ since reports on their immunomodulatory activities have mostly been confirmed at the level of innate immunity [46,47]. Their impact and importance to innate immunity is supported by their abundance in all eukaryotic organisms (fungi, algae, plants, invertebrate, and vertebrate animals), and distribution in cells and tissues at the front line of host defense against infection (mainly circulating immunocytes and epithelia) (see Figure 2). The dedicated Collection of Anti-Microbial Peptides (CAMP^R3^) database currently contains 8164 entries for peptides, most of which (~74%) come from animals [48]. A particularly abundant source are anuran species, with almost two thousand peptide sequences reported in the dedicated Database of Anuran Defense Peptides (DADP) [49]. AMPs are, however, also well represented in prokaryotes, produced by both Gram-negative and Gram-positive bacteria, with one abundant class being the bacteriocins [50]. In this case, however, their role is somewhat different to that in eukaryotes, as they are principally used to clear the immediate environment of producer bacteria from competition by closely related, bacterial strains [50].

## 3. Ribosomal vs. Non-ribosomal Synthesis and Antimicrobial Peptide Precursors

AMPs are often gene encoded and ribosomally synthesized (this is, by far, the most common case in eukaryotes), or can be assembled by large multi-functional enzymes known as non-ribosomal peptide synthetases (NRPSs) [51,52]. The latter process is used by bacteria and fungi [52] and allows incorporation of non-proteinogenic amino acids into the peptides (often the D-enantiomers of natural residues) and to further modify the peptides with ring formation, glycosylation, hydroxylation, or acylation [53,54]. There are ~500 non-proteinogenic amino acids known today, which possess added structural and functional features that can contribute significantly to a peptide’s potency. In fact, the cyclic peptide antibiotics polymyxin B, gramicidin S, and vancomycin are synthesized in this manner [54] and all contain some non-proteinogenic amino acids in their sequences [53,55]. 

**Figure 3:**
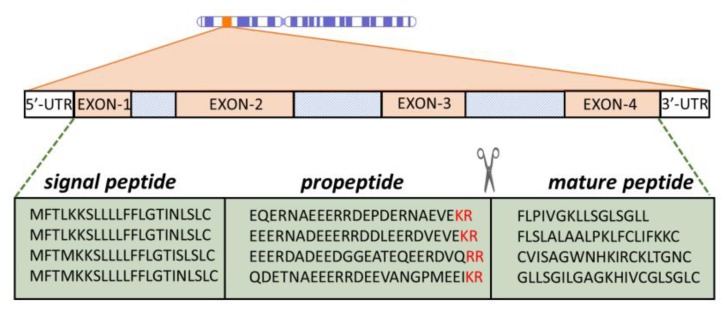
Representation of AMP expression. Peptides are cleaved at dibasic cleavage sites (-KR, -RR, in red). The proregion has a distinctly anionic nature. Depicted peptide sequences belong to anuran AMPs from the Ranidae family [60,61,62,63].

Gene encoded, ribosomally synthesized peptides are produced by almost all forms of life, including bacteria [42,48,56]. Quite often, multiple AMP genes are clustered at a single chromosomal locus, which is the case with α- and β-defensins [57] and can be co-expressed. They are, furthermore, frequently expressed as inactive precursors, containing a signal peptide region and a pro-piece that can serve to keep the mature peptide inactive until it is conveyed to the site of infection, where it is proteolytically released (see Figure 3). For this reason, the pro-piece is often anionic to complement the mature peptide, which is generally cationic. In most cases, the pro-region precedes (it is N-terminal to) the AMP sequence, but cases are known where it is the C-terminal ( such as for some fish and plant peptides) [58,59]. 

The activity of AMPS is, therefore, regulated not only by the expression level but also by the presence and abundance of appropriate proteases at the right place and the right time to cleave the peptide, which is generally at dibasic cleavage sites (see Figure 3) [57,64]. The signal peptide is a common feature of prokaryotic and eukaryotic proteins that need to enter secretory pathways [65]. A very useful aspect of antimicrobial pro-peptides is that signal regions for a given class can be much more evolutionarily conserved than the mature peptides themselves (see Figure 3) [58,66,67,68]. This provides a very useful handle for sequence mining in databases. The diversity of the mature AMP sequence most likely occurs as species’ adaptation to specific microbial communities in a particular environment. While there is still no solid explanation for the phenomenon of signal sequence conservation, it gives valuable insights into the evolution of some AMP families, as in the case with the anuran ones [69]. 

Lastly, it is worth noting that a majority of gene encoded AMPs undergo post-translational modifications, currently classified into more than 15 types, including disulfide-bridge formation, N-terminal or C-terminal capping (acetylation, pyroglutamic acid formation, amidation), halogenation, hydroxylation, phosphorylation, glycosylation, etc. Peptides can be modified to a greater or lesser extent, which contributes to peptide potency and/or stability [70]. 

## 4. Physico-Chemical Properties

Certain physico-chemical properties of AMPs are undeniably related to their interaction with lipid molecules that make up the bilayer system in the membrane of the target cells and correlate directly with the peptides’ biological activity and specificity. In fact, the same consideration could be made for other components of the microbial cell wall, but the data is less consistent. However, even limiting one’s focus to the lipid bilayer, there is still an imperfect understanding of the complex relationship between the AMP and membrane, since even peptides with very similar structures can have remarkably different reported mechanisms of action (e.g., buforin and magainin 2, or LL-37 and the rhesus orthologue RL-37) [71,72,73]. A better understanding of the relationship between physico-chemical properties and biological activity is, therefore, required to identify features that are responsible for potency and specificity of AMPs.

### 4.1. Molecular Type, Size, and Structure 

**Figure 4:**
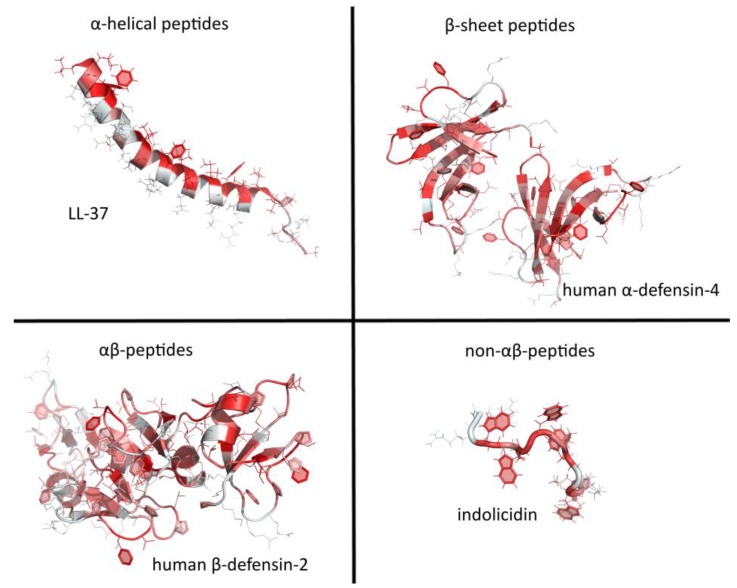
An overview of major structural classes of antimicrobial peptides. The structures taken as an example were solved either by nuclear magnetic resonance spectroscopy or X-ray diffraction and coordinates were downloaded from Protein Data Bank (PDB) (https://www.rcsb.org/) [77]. PDB IDs: LL-37 (2k6o), human α-defensin-4 (1zmm), human β-defensin-2 (1fd3), and indolicidin (1g89). Visualization was done using PyMOL 1.8 [78] and amino acids colored according to a normalized Eisenberg hydrophobicity scale (light grey—polar, red—hydrophobic) [79].

AMPs have been divided into several categories, according to particular features of their secondary structure. The simplest and most widely used classification divides them into extended structures, linear α-helical peptides and peptides with β-sheet or hairpin-like structures, generally braced by disulfide bridges [74]. Guha et al. [75] have, however, proposed a more elaborate taxonomy based on secondary motifs, with six different groups including *i*) α-helix, *ii*) 3/10 helix, *iii*) pi-helix, *iv*) β-strand, *v*) β-turn, and *vi*) disordered coil, which may concern the entire peptide or only parts of it, where some scaffolds combine to make different structural motifs. Essentially, this classification still depicts three major classes of AMPs: helical, β strand, and extended. In this review, the latest Wang terminology will be used to classify peptides in four classes [76]: α helical (e.g., human LL-37), β sheet (e.g., the human α defensin HD-4), αβ peptides (e.g., the human β defensin hBD-2), and non αβ peptides (e.g., the small, extended Trp-rich peptide indolicidin) (see Figure 4). 

The “α−helical” peptides, which are among the most studied and, consequently, the better understood, generally exhibit little or no structuring in a bulk aqueous solution, but only adopt this defined secondary structure in the presence of a bacterial membrane or some other type of anisotropic environment (sodium dodecyl sulfate micelles or water/trifluoroethanol mixtures) [46]. The adoption of this active structure is aided by:

- the presence of helix-stabilizing residues distributed throughout the sequence (e.g., Leu, Ala, Lys),
- the clustering hydrophobic residues on one side of the helix when it forms, which allows insertion into the membrane bilayer (or, seen the other way round, the presence of a lipid layer that induces appropriately distributed hydrophobic residues to cluster into a well-defined sector of the helix by interacting with it),
- salt-bridging between oppositely charged residues placed next to each other when the helix forms (normally, but not necessarily, when these residues are spaced three or four positions apart [80]).

It should be noted that increased helix stability correlates with increased potency, but only to a certain extent [81]. In fact, an increased propensity to transit from a coiled to helical conformation on passing from bulk solution to the membrane surface will generally positively affect activity. On the other hand, an increased propensity for helix formation in bulk solution will result in ‘sticky’ molecules (due to formation of a hydrophobic sector exposed to an aqueous environment), that tend to oligomerize or interact with other hydrophobic molecular surfaces. In the first case, this will alter the mode of the membrane interaction, which inevitably affects activity. In the second case, it leads to sequestration, with a negative effect on potency [72,82].

The size of helical peptides (sequence length) is also an important feature, especially in the context of peptide activity, since a minimum of seven to eight amino acids are needed to form an amphipathic structure [71] with separate hydrophobic and hydrophilic faces. One of the shortest peptides (only 10 residues) that was reported to have antibacterial activity, PGLa-H, was isolated from the skin of the African clawed frog *Xenopus laevis*, and is capable of adopting an α−helical conformation in an anisotropic environment [83]. Peptide length was initially thought to be particularly important when helical peptides were thought to form barrel-stave pores in the membrane (see below). At least 22 residues are required for them to span a canonical lipid bilayer, whereas the more extended peptides could do so with as few as eight residues [71]. However, very few AMPs have been found to form barrel-stave pores.

It is difficult to directly relate the length of a membrane-active peptide to its cytotoxicity. The bee venom-derived melittin becomes significantly less toxic when shortened from the original 26 residues to only the 15 C-terminal residues [84], but this is more likely related to the removal of a section with features favoring membrane insertion, rather than just reducing the size. In fact, by conversely doubling the size of the previously mentioned 10-residue PGLa-H in a tandem repeat resulted in a peptide with significantly greater potency against bacteria without greatly affecting toxicity toward host cells, so that the effect can be selective [85]. Therefore, it is wrong to conclude that the simple shortening or extending of a certain sequence will result in more favorable properties. It may be more appropriate to alter the length, while at the same time, maintaining an appropriate balance between hydrophobic and polar residues, and not drastically affect hydrophobicity or charge (see below). In any case, natural AMPs generally have relatively short sequences (normally under 50 residues) with the majority of known peptides in the 10-30 residue range [45]. 

With respect to other structural types, one well understood group are the proline-rich AMPs that, due to the abundance of proline residues, adopt an extended, likely polyproline type-II, conformation [86]. This type of AMP acts principally by translocating into bacterial cells without damaging their membranes (see below) using specific transport systems to then hit intracellular targets. Shortening these peptides affects both of these functionally essential aspects, and it has been reported that a minimum length is required for efficient antimicrobial activity [86]. In this case, requirements for the presence of specific motifs in the sequence may constrain how and to what extent a peptide can be shortened [87]. 

Trp-rich peptides, which are generally also Arg-rich, appear to enter bacteria via a sort of self-promoted uptake, without membrane disruption (see below), to then inactivate internal targets [88,89,90]. Trp residues are aromatic but the indole ring has a dual hydrophobic/hydrophilic nature and tends to partition at the membrane water interface. While the Arg residues undergo electrostatic interactions with membrane surface components, the guanidinium group also contributes to cell penetration. These distinctive properties make very short Trp-rich peptides active.

Defensins have size constraints that are determined by the fact that their scaffold is braced by at least three disulfide bonds, and participating Cys residues can be located towards the termini, so that shortening knocks bridges out with an inevitable effect on structure and function. In any case, some attempts have been made to design ‘mini-defensins’ by maintaining only part of the scaffold and only one disulfide bridge [91,92]. Therefore, appreciable antimicrobial activity is maintained. Several studies suggest that disulfide bridges are dispensable, so that linear analogues or fragments may appear to maintain or even increase activity with respect to the native peptides [92,93,94,95,96,97]. One study reported that the reducing environment of the colon may break disulphide bridges in hBD1, which renders it a more a potent antimicrobial agent [98]. This very likely alters the target and mode of action with respect to the native defensin, which is suggested by the fact that the IDR activity of defensins on host cells, which also likely depends on membrane interaction but in a different manner, can be significantly reduced in linearized peptides while the AMP activity is not [93,94]. Furthermore, oligomerization plays a significant role in the mode of action of many defensins, by determining how they interact with the membrane, and is likely to be very sensitive to such drastic structural alteration as knocking out disulfide bridges.

### 4.2. Charge and Hydrophobicity

The net charge of known natural AMPs varies widely from cationic (most often) to anionic (rare), which ranges from +16 to −6 [80,99,100,101]. The vast majority of identified peptides have an intermediate net positive charge (centering around +6) that can be directly correlated with peptide potency and selectivity. There seems to be an optimum charge span for activity, so that higher or lower values outside this range can result in reduced activity toward bacterial cells and/or increased toxicity toward host cells. The relationship between these parameters and function has again been most extensively probed in helical peptides. Dathe et al. [102] have shown that increasing the charge of magainin analogs above +5 resulted in both increased hemolysis and loss of antimicrobial potency. Giangaspero et al. [81] came to a similar conclusion when varying the charge of helical peptides, which, otherwise, had relatively similar mean hydrophobicity, amphipathicity, and helicity. The decreased amicrobial potency was, in part, attributed to a reduced propensity for helix formation due to the increased charge density (clustering of positive charges in one sector of the helix leads to repulsion). More recent findings suggest it could also result from repulsion between highly charged peptides at the membrane surface, which leads to a lower concentration of membrane-adsorbed peptides [103]. In principle, the distribution of positively charged residues should not correlate with peptide potency, which should only depend on the overall peptide charge [81,104]. However, it can make a significant difference if it affects the formation of helix-stabilizing salt bridges, as observed in artificial variants of the human helical peptide LL-37 [73]. 

In a similar way, there seems to be an optimal hydrophobicity window for peptides to have an optimal balance between antimicrobial activity and host cell toxicity. On average, AMPs contain approximately 50% hydrophobic residues. The overall hydrophobicity affects the peptide’s capacity to partition into the lipid bilayer, and can be directly correlated with both potency and host cell toxicity [105]. Increasing or decreasing this property outside the optimal range can result in a decrease of antimicrobial activity and an increase in blood cell lysis, not necessarily accompanied by improved antimicrobial activity [81,105,106]. In fact, an increased hydrophobicity can result in reduced antimicrobial activity if it promotes self-association, for the same reasons as an excessive stabilization of the helical structure (see above). This impedes access to the bacterial membrane and, therefore, lowers the concentration of the peptide actually impacting it [104,106].

### 4.3. Amphipathicity and Structural Stability in Helical Peptides

**Figure 5:**
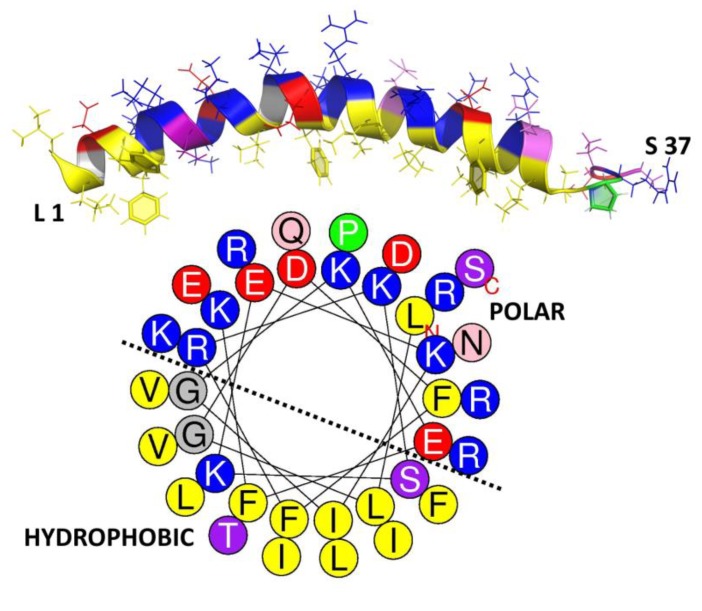
Secondary structure and helical wheel projection of human cathelicidin LL-37. The structure and projection were, respectively, obtained from PDB [77] (ID: 2k6o) and HeliQuest [109]. The residues were colored according to their hydrophobicity with ~40% hydrophobic and 60% polar amino acids in an appreciable amphipathic arrangement. Hydrophobic (yellow and green), polar charged [red (−) and blue (+)], polar uncharged (light to dark purple), and glycine (grey).

Overall, hydrophobicity is one of the key properties related to biological activity of any given AMP sequence. Unlike the charge, where its distribution is not necessarily correlated with potency (see above), the arrangement of polar and hydrophobic residues (~50% in AMPs) into an amphipathic structure sets some constraints on the primary structure, and it plays a key role in peptide activity more than the hydrophobicity itself [107]. However, what exactly is the amphipathicity? This property refers to the topographic distribution of hydrophobic and polar residues within the peptide sequence, which results in a more or less accentuated spatial separation in the active AMP structure. For a helical conformation, this occurs if polar/charged and hydrophobic residues cluster on opposite sides (of a hypothetical cylinder around which the helix is wound, see how this relates to the structure in the human helical AMP LL-37, in Figure 5). It can be numerically quantified in terms of the hydrophobic moment (μH) [79]. While an α-helix is one of the simplest and more efficient ways to generate an amphipathic structure, other types of active conformations such as in β−sheet peptides can also adopt an amphipathic arrangement, to a greater or lesser extent. However, it is more difficult to both visualize and quantify this [80,108].

Amphipathicity aids activity of helical peptides since it allows them to sink their hydrophobic faces into the membrane bilayer, which is an essential step leading to membrane disruption. It must be correctly tuned for an optimal balance between anti-bacterial potency and host cell toxicity. In general, the hydrophobic moment in helical AMPs is around 60% of the maximum possible value. Increasing it above this value does not greatly increase potency but can significantly increase toxicity [81,82].

Lastly, helicity is the propensity of an AMP to adopt a helical structure. As discussed above, it plays a significant role in the antibacterial activity, and, in general, it seems to correlate more with the toxicity toward host cells than antimicrobial potency, in a manner that relates to its effect on oligomerization. It can be reduced by incorporating *D*-amino acids into the peptide sequence, without greatly affecting potency. However, this can narrow the activity spectrum. As reported by Papo et al. [110], replacing up to a third of *L*-amino acids with their *D*-enantiomers resulted in peptides devoid of haemolytic activity that maintained an appreciable antibacterial potency, especially against Gram-negative bacteria. Furthermore, they are protected from proteolytic degradation, which should increase the bioavailability of such synthetic peptides.

## 5. Mode of Action

The mechanism of action of numerous AMPs has been extensively studied. Experiments have often been carried out with artificial membranes, typically large or giant unilamellar vesicles, and less frequently on microbial cells, using fluorescent dyes and labeled peptides. In any case, a widely accepted subdivision of AMPs, according to their mode of action, is *i*) membrane active and *ii*) non-lytic [111]. Some AMPs can act upon bacteria using both of these two major mechanisms, and sometimes switching from one to the other, depending on the peptide concentration, the membrane characteristics of a particular bacterial species, or its growth phase [112].

### 5.1. Disrupting Bacterial Cytoplasmic Membrane Integrity—A Primary Inactivation Mechanism

**Figure 6:**
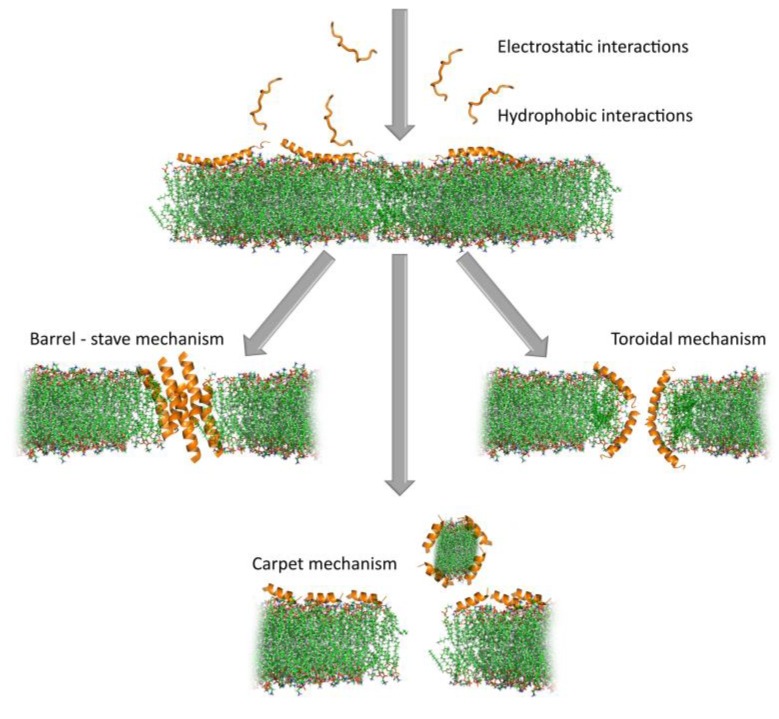
Proposed mechanisms of action of membrane permeabilizing peptides. Barrel-stave mechanism (alamethicin, PDB ID: 1amt), toroidal mechanism (magainin 2, PDB ID: 2 mag), carpet mechanism (aurein 1.2, PDB ID: 1vm5). Peptide structures were chosen from PDB [77] while taking their specific modes of action into account. The lipid bilayer was downloaded from the CHARMM-GUI.org website [120] (green: lipid tail, red, and blue: lipid head) and the manually created pores are only indicative. Visualization carried out using PyMOL 1.8 [78]. Note that not all interactions include pore formation and, for figure clarity, those are not included in this presentation (see above).

The term “membrane permeabilizing” peptides (MPP) [75] is more general than the often, and sometimes inappropriately used, “pore-forming” peptides [113,114,115]. Considering the complexity of lytic mechanism(s) of membrane-active AMPs (which is not restricted only to “pore-forming”), it is more appropriate (see Figure 6). An MPP must initially partition into a membrane and, therefore, be amphipathic for at least part of its structure, i.e., it must have some form of “interfacial activity.” This, nonetheless, allows for remarkable structural diversity, which results in functional diversity, so that some MPPs are active only against a narrow spectrum of microbial species while others have a very broad spectrum of activity. Wimley’s group has recently pointed out that the process of membrane permeabilization should not be considered as being simply due to a series of stochastic events involving one or more peptide molecules, nor should it be ascribed to a well-defined sequence of events. In other words, it requires neither discrete events nor the formation of static molecular entities. It is better described as a “mechanistic landscape” that varies depending on the experimental conditions and variables such as peptide concentration, bilayer lipid composition, temperature, ionic strength, and pH [75]. 

Results from a recent molecular dynamics (MD) simulation case study on maculatin, isolated from the skin glands of a green-eyed tree frog *Litoria genimaculata* [116], are in line with the proposed scenario. This particular peptide has been reported to act by pore formation [117]. Simulation work done by Wang et al. [118] showed that these pores continuously form and dissociate in the membrane. Moreover, the architecture of the pores varies, and is dominated by hexamers, heptamers, and octamers, with peptide molecules having a strong but not absolute preference for an antiparallel peptide orientation in the oligomers. Remarkably, the assembly of maculatin into pores seems to be driven by the successive addition of peptide molecules to an already existing transmembrane inserted helix to form a growing oligomer. Therefore, the translocation of the polar side chains of the incoming peptide is ‘catalyzed’ by the polar face of the already inserted peptide(s).

It follows that, even for very well-studied AMPs, the molecular mechanisms of membrane permeabilization cannot have been completely elucidated, and many questions remain [75,119]. Yet, some common steps can be inferred to occur at the bacterial membrane, which eventually leads to its disruption. This includes: i) initial attraction of the AMP to the membrane surface and interaction, ii) adoption of an active conformation, iii) insertion into the bilayer and concentration dependent accumulation, and iv) (in some cases) self-association/oligomerization [111]. The order in which these three steps occur will significantly affect the type of membrane lesion.

Most α−helical AMPs do not adopt this conformation in a bulk solution (see above) so that the initial interaction at the membrane level occurs between a positively charged peptide coil and the negatively charged phospholipid head-groups in the bacterial membrane surface [80,121]. This allows redistribution of hydrophobic sidechains so that they can interact with the membrane acyl chains leading to adsorption into the membrane, which, in turn, induces conformational changes in the peptide (to a stable amphipathic helix) that maximize these interactions [122], and this allows a deeper insertion into the lipid bilayer, which alters its structure. In other words, it follows the steps i), ii), iii), iv) in that order. By contrast, β−sheet peptides already have a stable, disulphide-braced amphipathic conformation that is maintained on membrane insertion. This is likely the case for pre-formed helical peptides, like LL-37, which are stabilized by internal salt-bridges [123]. In both cases, the pre-formed structures favor oligomerization [72,124], so these peptides may follow a different order for the steps ii), iv), i), iii). 

Once on the membrane, a critical peptide concentration is, in any case, required to induce membrane lysis, which can occur by different mechanisms (see Figure 6). Several different mechanisms have been proposed to lead to membrane lesions, which involve more or less well-defined molecular entities.

- Historically, the first proposed mechanism was for a certain number of peptide molecules to assemble and flip from a parallel to a perpendicular orientation with respect to the membrane surface, to form barrel-stave pores. The amphipathic structure would allow their hydrophobic surface to interact with the membrane lipids and hydrophilic regions to line the core of the channels, which promotes lateral peptide-peptide interactions. This mechanism, however, has turned out to be rare, and seems to apply to a limited number of AMPs, such as pardaxin [125] and non-proteinogenic alamethicin [126].
- In a second, less organized model, peptides remain aligned perpendicularly to the membrane surface, with the hydrophobic region inserted in among the acyl chains. On accumulation, this causes the bilayer itself to cavitate so that the hydrophilic region of the peptides line a wormhole or toroidal pore. Re-oriented phospholipid head groups also line the pore so that precise peptide-peptide interactions, or even a defined number of participating molecules, are not required, which makes it much more permissive for diverse primary structures than the barrel-stave pore. Such behavior is reported for the helical peptides magainin 2 [111] and aurein 2.2 [127]. These pores are reported to have relatively short lifetimes and can collapse, which allows the constituent AMPs to gather on the inside membrane bilayer surface, or it can extend and combine to lead to membrane micellization. For the bee toxin peptide melittin, for example, MD simulations suggest that toroidal pores are quite disordered and follow the latter pathway [128,129].
- In a third, even less organized model, peptides concentrate on and coat the surface to lead to micellar structures involving limited areas of the lipid bilayer, which, on removal from the membrane, leave large lesions behind. This non-specific, detergent-like mechanism does not necessarily require discrete pore formation but just surface accumulation, so it has been called the carpet model. It has been proposed alternatively for magainin and aurein 1.2 [130,131].

The last two mechanisms are not necessarily mutually exclusive, but could fit into Wimley’s “mechanistic landscape,” which occurs at different peptide concentrations, in a membrane-dependent manner. Other less disruptive mechanisms have also been proposed for AMPs, and include membrane thinning, depolarization or fusion, electroporation, and targeting of specific phospholipids [74,80,119]. In any case, they are all attempts to simplify mechanisms that are extremely complex and dependent on a number of variable conditions, in an attempt to make them more comprehensible. This poses the risk of limiting the mode of action of membrane permeabilizing peptides to a few “main categories” considered separately. But how realistic are these proposed models? And how reliably do they explain AMP behavior? Years of research carried out mostly on very simplified membrane models (e.g., liposomes) [75], have shown that the mode of action can vary substantially with very subtle modifications in a lipid-to-peptide ratio or membrane surface charge, even for a given AMP [132]. 

In summary, although we are far from a complete picture of how even the best studied peptides act, it is safe to say that AMPs, likely act in vivo using several possible membrane-disrupting mechanisms, with time frames and to extents that depend on environmental conditions. Any given permeabilizing model may, therefore, solve part of the puzzle but is unlikely because it does not provide the entire solution. This lack of dependence on specific interactors or defined mechanistic pathways may have contributed to the relatively low incidence of bacterial resistance to AMPs, despite many millions of years of continuous exposure to them. Bacteria can counteract them by altering the surface properties (mainly charge) in different ways, but this is a metabolically expensive, and, therefore, a transient form of induced resistance [133].

### 5.2. Non-Lytic Intracellular or Extracellular Mechanisms of Action

Some AMPs do not rely on a directly membranolytic mechanism, but act on extracellular or intracellular targets [74,86,112,134,135]. These act on the outside, disrupting septation or cell-wall biogenesis to impede cell division and weaken the structural integrity of the cell, or it can pass through the bacterial cytoplasmic membrane, without necessarily disrupting it, and inactivate specific metabolically essential components inside the cell.

With respect to the latter type, Trp-rich AMPs have been reported to enter bacteria by direct translocation, which is a process that has some aspects in common with pore formation, but without resulting in cell lysis [88,89,90]. Instead, the proline-rich AMPs enter susceptible bacterial cells using specific membrane transport proteins [86,136]. Even helical AMPs could, in principle, internalize into bacteria, without apparent membrane lysis, simply through the rapid formation/collapse of pores. Regardless of the peptide uptake mechanism, and according to a slightly modified Le at al. classification [135], internally and externally acting AMPs can be classified into six groups depending of their specific targets, which is listed below.

### 5.3. Nucleic Acid Biosynthesis and Metabolism Inhibitors

This group of peptides is represented by the helical buforin II and Trp-rich indolicidin [137,138]. Buforin I, which is the parent peptide to buforin II (a 21 amino acid fragment), is homologues to the N-terminal fragment of the DNA-binding protein histone H2A [139]. Some variants of buforin II have shown affinity toward double stranded nucleic acids [140], while designed analogues were found to have a greater binding affinity for RNA [141]. Indolicidin, which is a peptide of bovine origin belonging to the cathelicidin family, has been found to act both by disrupting the bacterial membrane and by inhibiting DNA synthesis, or, more specifically, inactivating DNA topoisomerase [138,142,143].

### 5.4. Inhibitors of Protein Biosynthesis and Folding 

Bovine cathelicidin Bac7, which is a 60 amino acid long peptide isolated from bovine neutrophils, interferes with complex machinery involved in protein synthesis. Its activity seems to reside at the N-terminus, so that a 35-long fragment, Bac7_1-35_, is fully active and has been shown to inhibit protein translation by targeting ribosome subunits, without affecting DNA synthesis or transcription. This specifically inhibits the process of protein synthesis [135,144,145,146,147]. Other proline-rich peptides with a similar mode of action include PR-39, the porcine orthologue [148], and unrelated apidaecin-type peptides isolated in honeybees, hornets, and wasps [149]. Apart from Pro-rich peptides, CP10A, which is a synthetic indolicidin derivative in which proline has been substituted with alanine, is an example of a short, tryptophan-rich helical peptide that, in addition to membranolytic properties, has DNA-binding affinities, and also acts by disrupting protein metabolism [150].

Proline-rich peptides are also reported to exert antimicrobial activities by interfering with protein-folding. Pyrrhocoricin, apidaecin, drosocin, and Bac71-35 all inhibit the major bacterial heat shock protein DnaK, and, in some cases, disrupt its ATPase activity [147,151,152,153]. They prevent DnaK from refolding misfolded proteins, and apidaecin has been shown to also inhibit the associated chaperonin GroEL [134,135]. Furthermore, these peptides bind stereospecifically to their bacterial target, which are inactive toward the human counterpart chaperone Hsp70 [151]. Other proline-rich AMPs with the same mode of action are the insect abaecin, and redesigned oncocin [135]. 

### 5.5. Inhibitors of Bacterial Proteases

Some AMPs, like histatin-5, have been reported to inhibit both host-secreted and bacterium-secreted proteases [154]. Dysregulation of these enzymes is associated with oral diseases such as periodontitis. By competitively inhibiting the bacterial cysteine proteinase clostripain, produced by *Clostridium histolyticum*, whose infections cause gas gangrene [155], histatins-5 and other peptides of this kind have been proposed as a potential therapeutic to reduce extracellular matrix degradation caused by bacterial or dysregulated host proteases. These AMPs reduce virulence and are antimicrobial.

### 5.6. Cell Division Inhibitors

CRAMP, the mouse helical cathelicidin orthologous to human LL-37, is a potent membranolytic expected of a helical AMP [156]. A CRAMP fragment has also been reported to interfere with the septation process, most likely by inhibiting bacterial cytokinesis [134]. This fragment was identified due to a significant sequence similarity to a bacterial peptide that regulates septation by interacting with its machinery [157]. C18G, which is a C-terminal, α-helical fragment of platelet factor IV, was also found to inhibit cell division by strongly stimulating the PhoQ/PhoP signaling system. This, in turn, results in increased synthesis of QueE, which is an enzyme that inhibits septation by interacting with the divisome [158]. 

Human α−defensin-5 targets different cell mechanisms, and also interferes with cell division processes, as shown by extensive elongation of peptide treated bacteria [159]. For similar reasons, AMPs of different origin such as bacterial microcin J25, insect diptericin, and the cathelicidins indolicidin and PR-39 have also been proposed to interfere with cell division processes [135]. However, the precise mechanism(s) involved have not been elucidated. 

### 5.7. Cell Wall Biosynthesis Inhibitors

The bacterial cell wall, being essential for the cell’s structural integrity and survival, is the target for numerous antibiotics in current use [160]. It consists of alternating β−1,4-linked N-acetylglucosamine and N-acetylmuramic acid crosslinked with peptide chains [161]. Lipid II is a crucial component of the cell wall synthesis process, since it is the shuttle carrier that transports disaccharide-pentapeptide building blocks across the membrane to be incorporated into the existing cell wall structure [162]. A number of AMPs, including the bacterial lantibiotic peptides mersacidin and nisin [163,164], and the fungal defensins plectasin and copsin [165,166] target this molecule in different ways, to disrupt cell-wall biogenesis. These AMPs, therefore, act externally to disrupt peptidoglycan integrity analogously to β−lactam antibiotics like penicillin or glycopeptides like vancomycin.

### 5.8. Lipopolysaccharide (LPS)-Binding Peptides

These peptides specifically act on this major structural and functional component of the outer membrane that covers the surface of Gram-negative bacteria. LPS can be released during bacterial cell division or death and induce a variety of inflammatory effects in animals, which leads to sepsis, and this may occur as a result of using antibiotics to treat Gram-negative infections. At the moment, there is limited treatment for patients with septic shock, which most often results in death [167,168]. AMPs that can bind to LPS may disrupt the outer membrane, which affects the cell’s structural integrity and reduces survival. They can make the cytoplasmic membrane more accessible to other AMPs/antibiotics that have difficulty in passing through the outer membrane, and can also help sequester and clear LPS, which reduces its pro-inflammatory effects.

On the other hand, the LPS layer can actively neutralize the activity of AMPs by inducing their self-association or aggregation and sequestering them [169]. This has been observed for the frog peptides temporins A and B from *Rana temporaria*. However, Rosenfeld et al. [170] showed a synergic effect between these peptides and temporin L, which prevents their LPS-mediated oligomerization and markedly improves their activity. Another way to restore activity is by introducing a boomerang motif (GWKRKRFG) at their C-terminus, which results in hybrid peptides no longer susceptible to LPS-induced aggregation [171]. Furthermore, melittin-cecropin hybrid peptide with two additional positive charges at the C-terminus proved to be effective in traversing the LPS layer [172]. In any case, amphipathicity and a high proportion of cationic residues in the AMP sequence seem to be important properties for the broad-spectrum LPS-binding peptides [173].

## 6. Strategies for Identifying or Designing New AMPs

### 6.1. Crude but Effective–Extraction and Assay-Guided Isolation

In the past, identification of novel AMPs involved handling of several specimens from the same species to obtain small amounts of active peptides. Initial tissue homogenization was followed by peptide extraction and the crude peptide was isolated in several steps, mainly by using chromatographic techniques. In some cases, the animals were pretreated with electric shocks or noradrenaline, or were exposed to bacterial infection, to stimulate AMP production [174,175]. Potential AMPs were then isolated by assay-guided fractionation and the sequence determined using different techniques, including Edman degradation and mass spectrometry. Magainin, which is one of the first frog peptides to be identified, was isolated in this manner, as were penaeidins, pleurocidin, and some mollusk cysteine-rich peptides, among others [176,177,178,179]. Several human peptides were also identified in this manner, from epithelial cells and plasma [180,181,182,183]. Some potential AMPs have been identified by analyzing lysates from proteins, and even common food sources, in particular whey. Theolier et al. [184] have recently reported six new peptide fragments from β-lactoglobulin and one fragment from α-lactalbumin derived by peptic cleavage of whey protein isolate, which all had antibacterial properties.

This approach is, therefore, evidently successful, but is also very time-consuming, and produces rather low yields. It can also raise ethical questions of animal protection, especially considering rare and endangered species. Lastly, it misses AMPs that are not constitutively produced or whose expression cannot be stimulated.

### 6.2. Make the Most of the Growing Abundance of Omics Data 

The rapid development and plummeting cost of sequencing techniques (next generation sequencing or NGS), combined with efficient and relatively cheap solid phase synthesis techniques, has opened the possibility of mining for valuable sequence information hidden in the genome, and functional testing, without the necessity of isolating polypeptides. For example, frog peptides have been identified by isolating total RNA and reverse transcribing the mRNA based on the 3′ poly-A tail. A cDNA library was constructed by using appropriate vectors and the positive clones selected and analyzed by nucleotide sequencing. This allowed the identification of several novel temporins, which were then either synthesized or obtained using “classical methods” including isolation and purification, before activity testing and confirmation using amino acid analyses [175]. A similar procedure led to the discovery of several peptides in the pickerel frog, *Rana palustris* [185], of clavanins from tunicate hemocytes [186], of protegrins from porcine leukocytes [187] and of penaeidins from Indian white shrimp *Fenneropenaeus indicus* [188], among others. More recently, *in silico* analyses of cDNA data in EST databases [189] led to the discovery of trichoplaxin, which is a placozoan AMP from *Trichoplax adhaerens* [190]. 

**Figure 7:**
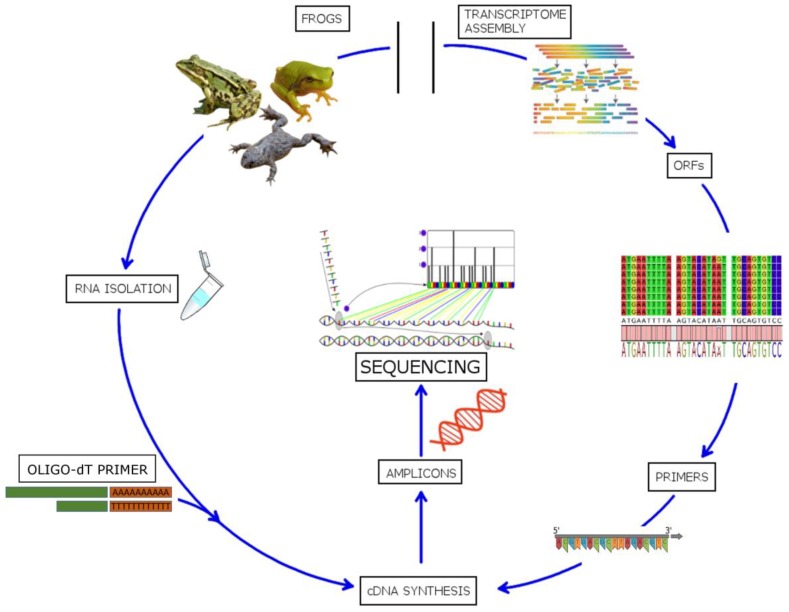
Schematic representation of the targeted DNA sequencing method. Figure modified from Rončević et al. [193] and reprinted under Creative Commons Attribution 4.0 International License (http://creativecommons.org/licenses/by/4.0/).

Improvement in NGS techniques and analysis pipelines, as well as the abundance of publicly available genomic and transcriptomic data, has led to the development of high-throughput techniques for simultaneous identification of potential AMPs. Kim et al. [191] reported a de novo transcriptome analysis of the American cockroach *Periplaneta americana,* which leads to the discovery of 86 putative antimicrobial peptides out of which 21 were experimentally verified for this activity. A similar approach was used for the identification of novel AMP sequences in the grasshopper *Oxya chinensis sinuosa* [192]. A novel method has recently been successfully developed for simultaneous identification of AMPs in different frog species [193]. By utilizing highly conserved signal regions of the peptide precursors (see Section 3) to design forward degenerate primers and correlating with transcriptomic and proteomic data available in public databases, ~130 different potential AMPs were identified, of which 29 were novel sequences (see Figure 7). The same procedure could, in principle, be applied to other organisms that have AMP gene families with comparable properties, i.e., a conserved signal peptide region associated with hypervariable mature peptide regions.

Brand et al. [194], on the other hand, developed a procedure for screening and identifying “intragenic antimicrobial peptides,” which are bioactive fragments from larger proteins, based on specific physico-chemical properties, by finding eight novel peptides with different antibacterial potency. This experimental method was developed to identify peptide sets with membranolytic effects in model membranes [195]. This is a complementary step to the previously developed bioinformatic pipeline. Yi et al. [196] screened seven previously assembled genomic and transcriptomic datasets in the amphibious mudskippers and, based on sequence similarity, identified ~500 novel peptide sequences with the correct characteristics, by opening new pathways for AMP discovery. A similar procedure led to the analysis of gill transcriptomes from 87 ray-finned fish species, which leads to the successful identification of some novel AMPs [197]. 

Another interesting approach, which combines “bioreactor” AMP synthesis and high-throughput sequencing, was reported by Tucker et al. [198]. This leads to the identification of several thousand potential AMP sequences. To this purpose, the Surface Localized Antimicrobial displaY (SLAY) techniques were developed, inducing bacteria to express and self-test a random 20mer peptide library constructed using a codons subset, which remain tethered to a protein on the bacterial membrane surface. Therefore, bacteria expressing bactericidal or bacteriostatic peptides are depleted from the population, so that a comparison of high-throughput DNA sequencing of plasmid libraries before and after induction of expression leads to the identification of potential antimicrobial hits. A similar technique for improving AMP potency had been proposed several years ago by researchers at Novozymes, termed the “suicide expression system” (SES), but for soluble peptides. This is a cis-acting system based on induced mutation of bacterially expressed, but tightly controlled peptides, that are then secreted in increasing amounts until they result in the death of the producer strain, and was adapted from previously reported systems [199]. A trans-acting peptide screening system (TAPS) was also developed in which peptides expressed and secreted by one bacterium are screened against other bacterial species. This type of system was used to optimize the sequence of the fungal defensin plectasin for development as an antibiotic [165,200].

### 6.3. Quantitative Structure-Activity Relationship (QSAR)—From Virtual to Novel AMPs

The above-mentioned methods have proven to be effective in identifying putative AMPs, sometimes suggesting several sequences to select from, but provide no indication as to the eventual potency of their activity toward bacteria or their toxicity toward host cells. Rational design of artificial peptides and redesign of natural peptides, based on various physico-chemical properties associated with potency and/or selectivity (e.g., net charge, amphipathicity, structuring propensity, tendency for self-aggregation, etc.), has, however, provided a body of data that can be used to predict functional characteristics from the sequence, especially, but not only, for linear helical AMPs [201,202]. 

**Figure 8:**
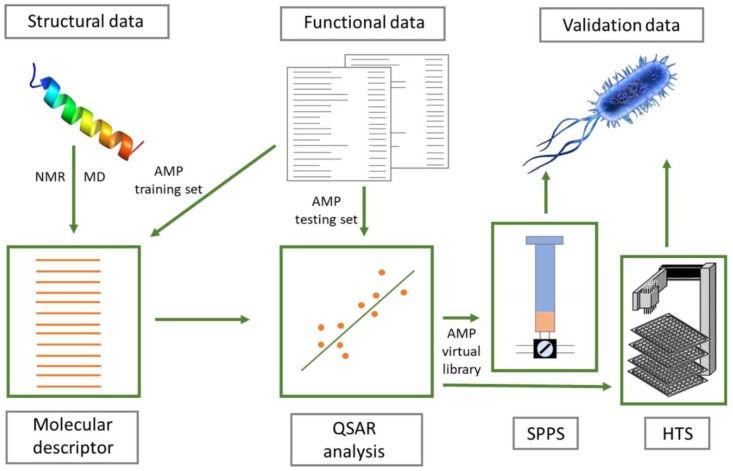
General overview of a QSAR method leading to design and validate novel AMPs. Structural data can be collected experimentally or predicted computationally (e.g., by MD). Functional data can be obtained from the literature or previous characterization campaigns to create specific databases. The best correlation between molecular descriptors and activity is determined based on statistical analysis, which allows us to propose new optimized sequences (putative AMP virtual library). These must then be either synthesized by solid phase peptide synthesis (SPPS) for in vitro validation and/or used for high-throughput screening (HTS) biological assays such as SLAY, SES, or TAPS (The 3D structure of magainin 2 was downloaded from PDB database (ID: 2 mag) and prepared using PyMOL 1.8).

QSAR approaches may include virtual screening studies where the biophysical properties of known active peptides are used to construct molecular descriptors that are associated with different functional aspects. These descriptors are then used to link a novel sequence to its likely biological activity [203]. The main assumption is that a mathematical function can be developed that correctly links physico-chemical properties (e.g., net charge and amphipathicity) with an observable outcome [e.g., minimal inhibitory concentration (MIC) values]. Typically, a number of molecular descriptors are created by linking physico-chemical properties with the biological effect in a training set of peptides where the former are measurable and the latter are experimentally determined. This is followed by a statistical analysis to determine which descriptor (combination of parameters) provides a predicted functional value that correlates best with the experimentally-determined values. The QSAR model is then validated on an external (testing) set of peptides (see Figure 8) [204,205].

Recurrent neural networks have successfully been used to develop such algorithms for de novo design of AMPs [206]. However, these models generate sequences without necessarily providing a quantitative prediction of antimicrobial activity. Witten and Witten [207] have improved on such models by creating a convolutional neural network, which was trained on a large set of peptides with known MIC values. This approach proved to be successful in designing AMPs, in which two have appreciable antimicrobial activity that has been experimentally validated.

An alternative approach is to combine QSAR with knowledge-based selection criteria for filtering putative AMP sequences. Adepantins were designed in this way, based on descriptors extracted from frog AMPs for which robust data on both MIC against *E. coli* and hemolytic activity were available. They proved to be remarkably selective toward some Gram-negative species [208]. The D-descriptor developed in this method has been recently adapted to the Mutator tool (http://split4.pmfst.hr/mutator/), which is a method that allows in silico re-design of peptide sequences to potentially improve selectivity. Dadapins were designed in this manner [209], by applying a strong filtering process on a devoted AMP database. In this case, we select for activity against Gram-positive bacteria, which is followed by optimization using the Mutator. They were shown to have high selectivity indices and comparable activities against Gram-positive and Gram-negative strains [210].

QSAR predictors are normally based on 2D-models, but, more recently, 3D-descriptors have also been developed [211,212]. This has, in part, been possible due to improved MD simulations on AMP/model membrane systems, which are used to optimize the starting 3D structure models, since experimentally determined 3D structures of AMPs are still rather limited [212].

### 6.4. MD Simulations—Seeing is Believing

This type of in silico approach has been used most often with helical, membrane–active AMPs [213,214], even though other types of membrane-active AMPs have also been studied [215,216]. Although they must always be considered critically and subsequently verified experimentally, MD simulations have the advantage of providing valuable information at the atomistic level, which is normally beyond experimental determination. This was the case for maculatin (see Section 5.1) where simulation data shed new light on the mode of action of this known lytic peptide [118]. Likewise, our group has recently used MD simulations to elucidate the mode of action of kiadins, which is another class of *in silico* designed peptides with membrane activity [217]. MD simulations can consider different trajectories to the membrane surface. For example, they can probe how the orientation of the peptide on its approach (e.g., how the angle of approach or peptide sector facing the membrane surface) affects the initial binding step, and subsequent insertion into the lipid bilayer (unpublished data). Simulation data can provide considerable insight on the mode of action of some peptides, which improves the existing models used for AMP prediction [218], but can also be used for a de novo design of peptides with desirable characteristics. Recently, an all-atom, simulation-guided design process, introduced by Chen et al. [219], has proven to be successful in producing a pore-forming AMP starting from a 14-residue polyleucine.

The main constraint with MD simulations is the computational time that is required to observe any given step in the permeabilization process [see steps i) to iv) in Section 5.1] [220,221]. Steps i) (binding) and iii) (insertion, but mostly in the specific conditions, such as at elevated temperature or when a pulling force is applied) are observed in a relatively short time-range. Therefore, these steps are amenable to an all-atom approach, but steps ii) and iv) (structuring and aggregation/pore-formation) can require considerably longer time frames and computational power to simulate. For this reason, simulations most often consider the AMP already in its active structure (which may not be realistic) and resorts to coarse-grain models when dealing with processes such as pore formation or translocation [222,223].

## 7. Therapeutic Potential

Since their initial discovery, AMPs have often been indicated as potential leads for the development of novel therapeutic agents for treating microbial infections. This has been one of the driving forces behind AMP research. However, the transition from in vitro to in vivo and translational applications of molecules derived from AMPs have proven to be very difficult [224,225]. Compared to “classical” antibiotic treatment, AMPs should have several advantages, especially when they are multimodal (can hit different microbial targets simultaneously), multifunctional (can directly inactivate microbes but also stimulate defense against them), fast acting, bactericidal, and can have accessory anti-inflammatory and/or healing activities [226]. However, they have evolved to act in a precisely orchestrated manner and are difficult to deliver as exogenous drugs. Furthermore, bacteria can fight back and interfere with AMP activity through proteolytic processing, active efflux, biofilm formation, and exopolymers entrapment, as well as by reducing their surface charge [227,228,229]. Especially worrisome is biofilm formation, since such bacteria can display up to 1000-fold higher resistance compared to planktonic bacteria due to the interaction of AMPs with specific components of the extracellular biofilm polymers [230]. Additionally, AMPs generally elicit relatively low levels of transient resistance compared to “classical” antibiotics. Several AMPs, such as the membrane-active gH625 and its analogue gH625-GCGKKKK, have been reported to be active against biofilm-forming bacteria [231]. In addition, Berditsch et al. [232] have recently reported a synergistic effect of two cyclic peptide antibiotics, polymyxin B and gramicidin S, against multidrug-resistant strains and biofilms of *Pseudomonas aeruginosa*. In this respect, AMPs can counteract biofilms in different ways such as by preventing their formation, and/or inactivating sessile bacteria, or modulating quorum sensing or twitching motility [233].

Despite years of trials, there are still some major obstacles to overcome before clinical application. Potent antimicrobial activity in AMPs is often accompanied by toxicity toward host cells. Although cationic AMPs preferentially target the negatively charged bacterial membrane, it has proven to be difficult to sufficiently reduce toxicity toward host cells, which can be significant, especially for helical AMPs. Melittin, for instance, is cytotoxic at comparable concentrations to those conferring antimicrobial activity [234]. These issues can, in principle, be resolved by designing peptides with more favorable physico-chemical properties (see above). Brevinin-1EMa analogues were less haemolytic when Ala residues replaced Leu, to reduce hydrophobicity [235]. A similar effect was observed when hydrophobicity was reduced at the N-terminus of mastoparan-X peptides [236]. In general, however, there is a trade-off between reduced toxicity and reduced potency. Other approaches used to reduce peptide toxicity include nanoencapsulation, which was the case with P34 [237]. It is also important to note that unwanted side effects may still be hidden, due to the large preponderance of in vitro over in vivo experiments involving AMPs. For example, it is well known that “classical” antibiotics impact gut microbiota, disturbing the well-adjusted and vital symbiosis between intestinal flora and the host [238]. It is likely that AMPs will display some toxicity toward the indigenous microflora, and partly disrupt its protective functions [239]. The host’s own AMPs likely play a significant role in maintaining the tightly regulated homeostasis of the microbiota, which exogenous AMPs could alter [233].

A second major concern is peptide stability under physiological conditions (i.e., in the presence of serum, salt, pH variations, and proteolytic enzymes), or rapid clearance, which can result in unfavorable pharmacokinetics. This is especially the case with linear peptides, which are easily attacked by host proteases and peptidases [239], and particularly problematic if the peptides are administered systemically [240]. For this reason, AMPs are predominantly being considered for topical applications. The stability can, however, be enhanced by peptide cyclization (linking the C- and N-terminus), which prevents the proteases interaction peptide due to steric hindrance, or introduction of *D*-isomers or unnatural amino acids into the peptide sequence, which makes it unfit for enzyme degradation [241]. However, this is expensive and precludes biosynthesis. Nanoencapsulation can improve peptide stability while, at the same time, reducing toxicity [237]. Another strategy is to PEGylate peptides (link them to polyethylene glycol), which has also been shown to increase bioavailability by reducing renal clearance [242]. Very recently, an interesting continuous subcutaneous delivery method has been tested for another proline-rich AMP, Api137, which shows that it improved efficacy in an in vivo model of infection [243].

Lastly, high manufacturing costs represent another major obstacle for wider use of peptide antibiotics [54,239]. Production of one gram of such a drug by means of solid phase chemical synthesis can cost several hundred dollars. Therefore, there is a need for less expensive production platforms such as, for instance, biosynthesis in fungal, bacterial, or even plant expression systems. In recent years, several attempts have been made in this field. However, none has hitherto proven to be commercially feasible. An exception is the fungal expression system used to successfully obtain sufficient amounts of plectasin for development [200]. 

Despite these obstacles, approximately 20 AMPs are at various stages of clinical trials at the moment, with the majority intended for topical applications [244]. They include cyclic and linear AMPs, such as a twelve residue histatin derivative, P-113, the magainin derivative MSI-78 (pexiganan, which failed to gain FDA approval for its original use), the twelve residue indolicidin derivative omiganan, the arenicin-3 analog AA139, the cyclic protegrin I analog murepavadin, and others [239,244]. There are still years of trials before such peptides are accepted for clinical use by governing agencies. It is, however, safe to say that AMPs remain one of the more promising compounds to provide new classes of antibiotics, despite a somewhat less pronounced enthusiasm than in the past. 

## 8. Conclusions

In the past 30 years, numerous studies have been carried out on AMPs, with most of them trying to answer to the question posed in the title of this review. In this case, it was our intention to focus not just on what we know, but also on some open issues surrounding the world of AMPs. It is hard to deny we are in the pre-post-antibiotic era, fully aware of the new, imminent threat to human health with no adequate solutions on the horizon. Can AMPs come, at least in part, to the rescue and justify years of motivated work by so many researchers? We are now well acquainted with how their structural and physico-chemical properties affect their activity but are still lacking a sufficiently deep knowledge of their modes of actions as well as the response of bacterial and host cells among them. This seems to be the major bottleneck in translating design and in vitro to in vivo efficacy as well as successful clinical applications. A limiting factor may be focusing too narrowly on pieces of the puzzle instead of adopting a wider perspective. Therefore, the question remains open—the potential is there but will we be able to tap it in a useful timeframe?

## Abbreviations

AMPs	antimicrobial peptides
FDA	Food and Drug Administration
HDP	host defense peptides
HTS	high-throughput screening
IDR	innate defense regulatory
MD	molecular dynamics
MPP	membrane permeabilizing peptides
NGS	next-generation sequencing
NRPSs	non-ribosomal peptide synthetases
PDB	Protein Data Bank
QSAR	quantitative structure-activity relationship
SPPS	solid phase peptide synthesis
SLAY	Surface Localized Antimicrobial displaY
SES	suicide expression system
TAPS	trans-acting peptide screening system
WHO	World Health Organization
